# Aggression and Helping as Responses to Same-Sex and Opposite-Sex Rejection in Men and Women

**DOI:** 10.1177/1474704918775253

**Published:** 2018-05-14

**Authors:** Joanna Rajchert, Karolina Konopka, Paweł Boguszewski

**Affiliations:** 1 The Maria Grzegorzewska University, Warsaw, Poland; 2 Nencki Institute of Experimental Biology, Warsaw, Poland

**Keywords:** aggression, helping, interpersonal rejection, sex differences, same-sex, opposite-sex

## Abstract

Research shows that interpersonal rejection increases aggression and decreases helping toward the rejecter. Based on the assumptions of the evolutionary approach, it was hypothesized that aggression would be higher and helping would be lower after rejection by a same-sex rather than an opposite-sex other. Moreover, it was predicted that the effect for aggression would be stronger in men, and the effect for helping would be stronger in women. Participants (*N* = 100) were rejected or accepted by a same- or opposite-sex person, and later aggression and helping were measured using the tangram Help-Hurt task. The major finding was that same-sex rejection resulted in more aggression and less helping than opposite-sex rejection, but the rejectee’s sex did not moderate the effect. Instead, men were more aggressive and less helping independently of condition. Along with the sexual exchange theory, more negative behavior in same-sex rejection could be interpreted as raised in-group sexual competitive tendencies, whereas less negative behavior in opposite-sex rejection could result from the motivation to exchange resources between men and women.

Experimental research on the effects of rejection is usually conducted in the same-sex or unknown-sex condition; the results suggest that exclusion increases negative affect and decreases positive affect and threatens belonging and control (Gerber & Wheeler, 2009). Some researchers also argue that, although rejection causes a shift toward a less positive state, it does not induce distress because participants in general declare a neutral or mildly positive state after rejection (Baumeister, DeWall, & Vohs, 2009; Blackhart, Nelson, Knowles, & Baumeister, 2009). Nevertheless, most of the research indicates that, compared to acceptance, rejection is followed by more aggressive (e.g., Buckley, Winkel, & Leary, 2004; DeBono, Layton, Freeman, & Muraven, 2017; Twenge, Baumeister, Tice, & Stucke, 2001; Warburton, Williams, & Cairns, 2006; for a review, see Gerber & Wheeler, 2009; Leary, Twenge, & Quinlivan, 2006) and less helping behavior (Twenge, Baumeister, DeWall, Ciarocco, & Bartels, 2007). However, there is also a possibility of more positive behavior after rejection when there are opportunities to regain the inclusion status in future interactions with the rejecter (Maner, De Wall, Baumeister, & Schaller, 2007).

Although much is already known about affective and behavioral responses to rejection by a same-sex or unknown-sex other, studies directly addressing the difference in reactions to same-sex and opposite-sex rejection are lacking. There is a study showing higher distress after same-sex rejection than after opposite-sex rejection (Bolling, Pelphrey, & Vander Wyk, 2012); however, it does not inform predictions regarding behavior. When behavior is considered, men are generally less aggressive toward romantic female partners than toward same-sex nonintimates, while that pattern of results is reversed for women (Bates, Archer, & Graham-Kevan, 2017; Bates, Graham-Kevan, & Archer, 2014). Although referring to behavior, these studies do not allow for determining which of the two factors affects aggression—intimacy or the sex composition of the relationship, or both. There are, however, studies (Cross & Campbell, 2012; Cross, Tee, & Campbell, 2011) that controlled for intimacy and showed that men as well as women show higher aggression in same-sex than in opposite-sex relationships, but that women are more aggressive in intimate relationships. Still, the reviewed studies do not show sex differences in same-sex and opposite-sex situations after rejection but only in neutral situations. The aim of the study reported in the present article was thus 2-fold. First, we wanted to test whether there would be differences in aggressive and helping responses to same- versus opposite-sex rejection. Second, we aimed at ascertaining whether sex differences influence aggressive and helping behavior after rejection by a same-sex or opposite-sex peer. Additionally, we were also interested in how same- and opposite-sex rejection would influence the affect and needs of the rejectee. We found an evolutionary approach to aggression and rejection to be a good basis for stating predictions.

## Evolutionary Approach to Same- and Opposite-Sex Behavior

From an evolutionary perspective, in contrast to social learning theories (e.g., the general aggression model; Anderson & Bushman, 2002), a moderate amount of aggression could be an adaptive behavior. Ferguson and Beaver (2009) propose that aggression is a “behavior which is intended to increase the social dominance of the organism relative to the dominance position of other organisms” (p. 287). As they argue, dominance leads to reproductive success, and thus, aggression is adaptive. Although they observe that aggressive behavior risks retaliation, injury, disapproval, or other adverse outcomes, in some situations, the risk of not being aggressive can be higher than that of being aggressive. Ferguson and Beaver (2009) further posit, following Gottschalk and Ellis’s (2009) summary of evolutionary concepts regarding violence, that males engage in greater levels of aggression because of greater sexual competition. Sexual selection theory argues that when making mating decisions, both sexes consider costs and benefits of the choice. Trivers (1972) argued that the parental investment (time, effort, and resources) that each sex must make in order to produce and nurture an offspring is one of the most important factors that is taken into account in those calculations. The sex that invests the least will tend to favor quantity over quality of mates and will compete with same-sex rivals more. Men’s parental investment is always much more limited than women’s because women must bear the costs of pregnancy and thus will be more selective in seeking possible mates. Therefore, it can be predicted that men will be more aggressive toward same-sex targets because aggression can increase dominance over other men, enabling greater access to mates. In the case of women, because they invest in the care of the young (including pregnancy), they in general will not be interested in having many mates and thus will compete less and will be less aggressive toward other women. However, women exert a great deal of selective pressure over men in the traits they look for when selecting mates because they are interested in quality of a mate. Those traits should increase the survival prospects of their children. Women select men who possess traits that would provide resources and assistance. Thus, based on Gottschalk and Ellis’s (2009) assumptions, men should be less aggressive and probably also more prosocial toward women than toward men. Considering this theoretical argument, women’s aggression should be lower than men’s, but the question remains—would it be equally low in same- and opposite-sex contexts?

Additional information on same- and opposite-sex behavior of men and women comes from the sexual exchange theory of Baumeister and Vohs (2004), which also refers to sexual selection but emphasizes that women are in control of an important resource: sex. This theory is inspired by social exchange theory, which allows for predicting the behavior of two parties in their interaction on the basis of costs and benefits specific to each of them. Sexual exchange theory assumes that sex is a women’s resource and that men are buyers of sex. Sex might be a women’s resource mainly because, as Trivers (1972) proposed, men’s investment in parenthood could be minimal while women’s is always substantial. The potential costs inhibit women’s sexual behavior. Thus, to induce women to have sex, men must offer some resource that women want. This resource might be commitment, belonging, or care, which would be very helpful when women become pregnant or have children. However, the theory of sexual economics posits that the price of sex will depend on supply, demand, competition among sellers, and other market factors. In this context, inclusion and exclusion by a same-sex or opposite-sex person might signal different things and might influence the price of sex and in consequence people’s behavior.

## Aggression and Helping as Strategies of Dealing With Same-Sex But Not Opposite-Sex Rejection

In general, exclusion in the evolutionary past threatened survival, and being repulsed from a group could have detrimental effects for both men and women (Baumeister & Leary, 1995). Kurzban and Leary (2001) propose that rejection in a broad sense is an adaptation for sociality—a kind of constraint that directs social effort in productive ways. Exclusion is an adaptation that allows for avoiding interactions with individuals who are poor partners for social exchange (who create greater costs than benefits). Examples of social exclusion in nonhuman animals are territoriality (exclusion of other individuals from a particular area) and status hierarchies (organisms at the top impose restrictions on those at the bottom). Kurzban and Leary (2001) argue that especially the latter parallels the phenomenon in humans. Thus, aggression in this context, defined by Ferguson and Beaver (2009) as a strategy used to gain dominance, could be considered the most obvious way of improving social status after rejection. However, it should be more probable after same-sex rejection, and more among men than among women, as aggression is a risky strategy of gaining dominance.

It would, however, be too simplistic to see aggression as the only strategy for rebuilding dominance. Hawley (1999) defines dominance as differential ability to control resources—without reference to how this is done. Also in this vein, increased or decreased prosocial behavior in certain situations could help to achieve domination. And indeed, as was already indicated, aggression (e.g., Twenge et al., 2001) and inhibited helping (Twenge et al., 2007) are the most common reactions to same-sex rejection, at least by men, while ingratiation (Romero-Canyas et al., 2010) or prosocial behavior (Maner et al., 2007) in such situations are dependent on many additional factors.

Despite the plausibility of the above argument for why same-sex aggression is greater in men than women, if sexual economics rules apply, women also compete for men’s resources. Rejection by another woman may, as in men, signal low social status, which may result in limited access to the highest ranked men offering the best resources. As aggression has a potentially high cost, inhibition of helping might be a more advantageous strategy for rebuilding social status in women than aggression. It has been shown that women threatened with exclusion by other women engage in exclusion more than men (Benenson et al., 2013; Benenson, Markovits, Thompson, & Wrangham, 2011). The authors argue that exclusion is women’s primary strategy of competing, whereas men might prefer more direct forms of aggression. Thus, not helping the same-sex rejecter, which might be a less direct way of dominance rebuilding, would better suit women’s adaptation. Also, Gerber and Wheeler (2009) proposed that inhibition of helping after social exclusion may serve the same function as aggressive behavior, namely, rebuilding the thwarted control need, which might be related to lower dominance. However, most of the studies have shown no sex differences in behavioral reactions to rejection in a same-sex relationship (Buckley et al., 2004; Twenge et al., 2007) or when the sex of the rejecter was unknown (e.g., Ayduk, Gyurak, & Luerssen, 2008; Warburton et al., 2006). Since sex differences were not always analyzed (e.g., Twenge et al., 2001) and in some research participants were predominantly women (e.g., DeWall, Twenge, Bushman, Im, & Williams, 2010), these results are not conclusive.

Regarding rejection by an opposite-sex other, aggression or not helping as strategies to gain dominance would not be as useful as in the case of same-sex rejection. Rather, being rejected by a man could be information for a woman that the market value of her sexual offer is low. This could lower the woman’s expectations regarding the value of the offer placed by the rejecting man or men in general. Also in line with the sexual exchange theory, women rejected by men could be ready to exchange sex for less. If the resource women strive for is a man’s commitment or investment, then they would be more inclined to get it in any possible way. On the other hand, rejection by a woman suggests that a male must offer more valued resources to get what he wants. Thus, male rejection of a woman might result in her being less selective when choosing a mate and opting for more short-term sexual relationships. Female rejection for most men could result in more ingratiating or at least less negative behavior toward women.

This line of thinking was partially tested in women and in men. The test, however, referred to inclusion or exclusion by other people in general. A team of researchers showed that, first, when people were convinced that they would have many satisfying friendships in the future or when they recalled instances of being accepted by others, both men and women then indicated higher interest in mating (Brown, Young, Sacco, Bernstein, & Claypool, 2009). The argumentation behind this finding is that people are motivated to mate when their survival is not threatened and that being rejected is a survival-related cue (as living in a group provides protection and resources). Furthermore, Sacco, Brown, Young, Burnstein, and Hugenberg (2011), based on the same assumption that mating goals are generally delayed until survival goals are met, indicated that inclusion facilitates more risky sexual strategies (e.g., mate poaching) than control or exclusion conditions in men, but not in women, because those strategies are adaptive for men. Although these authors did not refer directly to the sexual exchange theory when explaining the results of that study, it is probable that inclusion, in contrast to neutral or exclusion conditions, signals that men do not need to offer more or better resources to gain sex. Furthermore, Sacco, Young, Brown, Burnstein, and Hugenberg (2012) showed that women, after being told that they would not have any satisfying relationships in the future or when they recalled a rejection situation from their lives, might seek reaffiliation with men through engaging in more short-term mating strategies. As rejection in the past might easily threaten survival, especially for women who are taking care of children, affiliation is an important resource that women need for their protection and that men can offer in exchange for sex. Also, Buunk and Massar (2012) conducted a study in which participants had to divide resources between themselves and either a same-sex or an opposite-sex other. Results showed that men chose competitive resource allocation more often in the same-sex condition, but women were equally competitive toward men and women. This study also indicated that men were more prosocial toward women than women were toward men. The results confirm that, in the neutral situation, men might be more willing to offer resources to women than women are to men. At the same time, men tend to compete with other men, which might be seen as a consequence of men’s mating strategy favoring quantity of mates.

Although these results conform to sexual exchange theory, they do not show differences in response to same-sex and opposite-sex rejection in men and women. The current study directly tested those differences with an array of responses—positive and negative affect, belonging and control needs, and behavior: aggression and helping.

## Current Study

The aim of the present study was to test sex differences in threat to needs, emotions, and behavior after exclusion, in relation to the sex of the rejecter. We based our predictions mostly on assumptions derived from the sexual exchange theory but also the evolutionary approach to aggression which defines aggression as a means to increase an organism’s social dominance and puts greater emphasis on selective pressure in males’ aggression. We also assumed that rejection is a survival-related cue, and as such results in distress manifested by a drop in positive affect, belonging, and control and an increase in negative affect, as other studies have found (for a meta-analytic review, see Gerber & Wheeler, 2009, or Blackhart et al., 2009). As most of the previous research on social exclusion did not reveal sex differences, Williams (2009), in his temporal need threat model of social exclusion, argues that instant response to rejection is universally automatic and painful. As belonging is one of the most important factors in the survival of a human being, its loss should alarm the individual and the distress should be strong and general. However, the previously mentioned study by Bolling et al. (2012) showed that distress was higher in same-sex conditions than in opposite-sex conditions because being excluded by an in-group (same-sex other) could be more painful than being excluded by an out-group (opposite-sex). So, although we predicted that distress would be higher in the rejection than in the inclusion condition, both among men and women, we also predicted that same-sex rejection would result in more distress than opposite-sex rejection.

Behavioral reactions to rejection should generally follow affective reactions and may involve efforts to cope with it. Aggression (direct or indirect) as well as helping (or not helping) as strategies for gaining a competitive advantage were already proposed by Archer and Coyne (2005), Campbell (1999), and Hawley (1999). Given such strategic considerations, behavioral reactions to rejection will likely depend on situational factors such as whether it is a same-sex or opposite-sex rejection. In line with the evolutionary approach to social rejection (Kurzban & Leary, 2001) and the sexual exchange theory (Baumeister & Vohs, 2004), we predicted that same-sex rejection would constitute a threat to social status that could hurt chances for mating in both men and women. Women low in the social status might have smaller chances in sexual competition with other women for men offering better resources, and men low in social status would not have enough resources to ensure mating. Thus, although aggression toward same-sex others might be a risky strategy, potential benefits in the form of increased status could be worth the risks of such a strategy. Also, not helping a same-sex rejecter could be a good and safer (less costly) strategy for gaining an advantage over him or her. In the case of opposite-sex rejection, however, aggression or not giving help to the possible mating partner would be less functional. Moreover, rejection by the opposite-sex other might signal low sexual value on the part of the rejected individual, which could lower the woman’s price of sex and increase men’s offer of resources. Thus, we predicted that people would be less likely to engage in aggressive behavior and lack of helpfulness toward opposite-sex rather than same-sex rejecters.

However, as discussed earlier, there is a theoretical basis for expecting that behavior after same- and opposite-sex rejection will be different for men and women, and past research seems to support that prediction. In neutral situations, men tend to be more aggressive than women regarding physical and verbal aggression, but women show more relational and indirect forms of aggression than men (Archer, 2004; Bettencourt & Kernahan, 1997; Bettencourt & Miller, 1996). Other research has shown that same-sex rejection decreases helping and increases aggression in men and women to an equal extent (Buckley et al., 2004; Twenge et al., 2007). Those studies, however, did not allow for choosing a strategy of coping and measured only aggression or helping. As there was no other way to rebuild dominance, both men and women used only one given behavioral option. Considering previous results and theoretical assumptions, we predicted that, since an aggressive strategy of coping with rejection is rather more adaptive for men and less direct aggression is more adaptive for women, aggression would be higher and helping would be lower in the same-sex rejection than in the opposite-sex rejection and inclusion conditions, but the effect for aggression would be stronger in men and the effect for helping would be stronger in women.

Summarizing, we predicted that (1) distress would be higher in the rejection than in the inclusion condition, both among men and women, but same-sex rejection would result in more distress than opposite-sex rejection and (2) aggression would be higher and helping would be lower in the same-sex rejection than in the opposite-sex rejection and inclusion conditions, but the effect for aggression would be stronger in men and the effect for withdrawal of helping would be stronger in women. To test these predictions, a study was conducted in a 3 (same-sex rejection, opposite-sex rejection, and acceptance) × 2 (man and woman) design.

## Method

### Participants

Participants were 100 students (54 women), ages 18 to 20 years (*M* = 18.35, *SD* = 0.65), who volunteered for the study. Each participant gave his or her written consent to participate in the study, and the procedure was approved by the ethical committee. Based on the effect sizes obtained in other studies on effects of exclusion on behavior, the number of participants per cell was established to be at least 13 (*d* > 1.00; based on Gerber & Wheeler, 2009) to obtain a satisfactory level of statistical power equal to .80 (α = .05). In this study, there were 29 participants in the acceptance condition, 35 in the same-sex rejection condition, and 36 in the opposite-sex rejection condition, with a similar number of men and women in each condition.

### Materials and Procedure

Participants were invited to take part in an experiment on building relationships in pairs online and learned that they would be paired with another person with whom they would first be interacting and solving tasks online and would later meet face-to-face. Participants came to the lab individually and sat in front of a computer screen. All instructions and tasks were then presented via computer. The computer program used to conduct the experiment, as well as the whole protocol for the study, are available for free download (http://www.pmbogusz.net/?a=behapresenter). The study can be easily replicated with the use of this computerized protocol.

Participants first described how they felt by responding to statements such as “Now I feel good” on a scale from 1 (*not at all like that*) to 7 (*very much like that*). There were 13 statements measuring mood, including general mood (feel good), sadness, happiness, irritation, joy, anger, contentment, rage, tension, hurt, excitement, dejection, and calmness. Each statement was presented separately and was written in the center of the screen with numbers from 1 to 7 presented below it. Following the mood description, participants in the rejection condition gave information about their sex and received the information about the sex of their interaction partner, which was randomly assigned. Participants in the acceptance condition did not receive any information on the partner’s sex, making it a control condition. Next, we informed participants that they would be answering questions about their opinions, preferences, and interests by choosing one answer out of the four to each question. Each question and chosen answer was visible on demand to their online partners sitting in another room, who could use their answers to get to know them. Participants then chose answers to 12 questions based on the small-talk set of questions used in the Aron, Melinat, Aron, Vallone, and Bator (1997) study.

After participants chose an answer to each question, information was presented in the center of the screen saying that “the partner was interested in your answer.” The time set for “waiting” differed from answer to answer but was the same in all conditions and ranged from 2 to 6 s. Following that, participants were asked if they “want to continue the interaction with the allocated pair partner,” and after giving their answer, they saw the answer to the same question that their partner gave (answers were to be visible for the partners at the same time). In the acceptance condition, participants learned that their partner wanted to continue the interaction. In the rejection condition, the partner did not want to continue the interaction. Participants then answered need-satisfaction questions and described their emotions in the same way as before the manipulation. The needs were measured through three questions which addressed belonging (I feel accepted/rejected/ignored by my partner) and three questions which addressed control (“I feel that for my partner my opinion counts,” “I feel I have an influence on my partner’s decisions which refer to me,” “I feel I have control over what is happening between me and my partner”).

After participants described their needs and feelings, the measure of hurting and helping, a new procedure developed by Saleem, Anderson, and Barlett (2015) called the tangram Help-Hurt task, was administered. The tangram Help-Hurt task allows helping and aggression to be measured within the same procedure and gives participants the opportunity to engage in both aggression and helpfulness but also to avoid both behaviors. Participants in the Help-Hurt Task are informed that they will be building tangrams—puzzles constructed from geometric shapes—which will be assigned to them by their partner, after they first choose puzzles for their partner. Participants are told that they will get a reward (in our study, it was a cinema ticket) if they manage to complete 10 of the 11 tangrams selected by their partner within 10 min. Participants choose tangrams to assign to their experimental partner from a set of 30 in which 10 tangrams are labeled “hard,” 10 are labeled “medium,” and 10 “easy.” Because the partner could skip 1 of the 11 assigned tangrams and still get the reward, the index of aggression (hard tangrams) and helping (easy tangrams) was the number of tangrams in each category greater than 1. Participants can avoid helping or hurting by allocating to the partner the tangrams with medium difficulty level. In the validation studies, helping and aggression scores were correlated (*r* = −.73) but were not a simple inverse of one another (Saleem, Anderson, & Barlett, 2015).

After aggression and helping measurement, participants indicated their motivation to help or hurt their partner by responding to two questions (“I wanted to help my partner to earn the reward” and “I wanted to make it difficult for my partner to earn the reward”) using a 5-point Likert-type scale (1 = *strongly disagree* to 5 = *strongly agree*). After completing the assessment, participants were asked what they thought about the study and whether they had any questions, and they were debriefed and thanked.

### Results of Preliminary Study Testing the Effectiveness of the Rejection Procedure

Because a new procedure of rejection manipulation was used in this study, first a preliminary test of the procedure was conducted in a 3 (rejection, acceptance, and no-feedback conditions) × 2 (man and woman) design. We wanted to verify whether the rejection manipulation lowers satisfaction of belonging and control needs and increases aggression compared to an acceptance or no-feedback condition.

Participants were 49 students (23 men and 26 women, ages 19 to 30 years, *M* = 22.30, *SD* = 2.38). The measures and procedure that we used were as described in the Method section, with two exceptions: The sex of the rejecter was not revealed and the no-feedback control conditions were added (participants received information that the partner was not interested in their answers and did not give feedback regarding inclusion or exclusion on time). Results showed that the effect of conditions for belonging was significant, *F*(2, 43) = 6.55, *p* = .003, η^2^ = .23 but not moderated by sex. A Bonferroni post hoc test showed that belonging was higher in the acceptance than in the rejection condition, *p* = .022, and in the no-feedback condition, *p* = .004, but was not different between the rejection and no-feedback conditions. The effects of conditions for control and affect were not significant. However, the results of a multivariate analysis of variance (MANOVA) showed that the manipulation significantly affected behavior, Wilks’ λ = 0.702, *F*(4, 84) = 4.36, *p* = .005, $\eta_{\text{p}}^{2}$ = .16. Specifically, exclusion influenced aggression, *F*(2, 43) = 4.83, *p* = .013, $\eta_{\text{p}}^{2}$ = .18, and helping, *F*(2, 43) = 8.99, *p* = .001, $\eta_{\text{p}}^{2}$ = .29. The Bonferroni post hoc tests for helping demonstrated that helping was lower after rejection, *M* = 2.00, *SD* = 1.73, than after acceptance, *M* = 4.93, *SD* = 3.35, *p* = .010, or after no response, *M* = 5.75, *SD* = 2.48, *p* = .001. Aggression was higher in the rejection condition, *M* = 3.23, *SD* = 2.68, than in the no-response condition, *M* = 0.87, *SD* = 1.20, *p* = .012, but was not significantly higher than in the acceptance condition when Bonferroni correction for multiple comparisons was applied; otherwise, it was significant, *p* = .044, *M* =1.62, *SD* = 2.30. Sex of participants did not change the effects of conditions for behavior. As in previous studies, when compared to acceptance or no-response, rejection lowered helping given to the rejecter (Maner at al., 2007; Twenge, et al., 2007). Rejection also increased aggression compared to acceptance, but more so, compared to the no-feedback condition. Thus, we considered the rejection manipulation procedure of inclusion and exclusion valid and ready to use in our study.

## Results

First, indices of positive affect (PA) and negative affect (NA) and needs were created by factor analysis conducted using a maximum likelihood method of factor extraction with a Promax rotation. The results of the factor analysis were as expected, with items referring to PA (feel good, joy, happiness, contentedness, excitement, calmness, Cronbach’s α = .76 for PA1—before manipulation, and .88 for PA2—after manipulation) and NA (rage, hurt, tension, anger, sadness, dejection, irritation, α = .77 for NA1, and .93 for NA2). Belonging (α = .65) and control (α = .77) also loaded on separate factors. The observations for affect and needs were averaged.

Also, before testing the hypothesis, the number of allocated difficult and easy tangrams was analyzed to establish whether this was related to the motivation to help or hurt the rejecter. The zero-order Pearson correlation indicated that the number of difficult tangrams allocated to the partner was positively related to the intention to hurt the rejecter’s chances for reward, *r* = .75, *p* < .001, and negatively to the motivation to help the rejecter to win the reward, *r* = −.41, *p* < .001. The number of easy tangrams chosen by the participant was positively associated with the intent to help, *r* = .44, *p* < .001, and negatively with the motivation to hurt the partner’s chances for reward, *r* = −.68, *p* < .001. Thus, the number of difficult tangrams (larger than 1) allocated to the rejecter could be interpreted as a measure of aggression and the number of easy tangrams as a measure of helping. Aggression and helping scores were highly correlated, *r* = −.80, *p* < .001.

First, the hypothesis regarding changes in distress (affect and needs: belonging and control) was tested. Because belonging and control needs were not significantly correlated (*r* = .18, *p* > .06), separate two-way analyses of variances (ANOVAs) were conducted with a 3 (same-sex rejection, opposite-sex rejection, and acceptance) × 2 (man and woman) design to assess the effect of the manipulation and participant’s sex on needs satisfaction. Considering belonging, the results showed no interaction effect (*F* < 0.81, *p* > .45) or main effect of participants’ sex (*F* < 0.11, *p* > .79). Only the main effect of exclusion manipulation was significant, *F*(2, 94) = 29.80, *p* < .001, $\eta_{\text{p}}^{2}$ = .39. Belonging was significantly higher in the acceptance condition than in the same-sex (*p* < .001) and opposite-sex (*p* < .001) rejection conditions, but the same-sex and opposite-sex rejection conditions did not differ significantly (*p* > .09). The results for the control condition showed no significant main effect of condition (*p* > .94), no main effect of participants’ sex (*p* > .77), and no significant interaction of those variables (*p* > .11).

To test the differences in affect due to the participants’ sex and conditions, a repeated measures ANOVA with one independent within-subjects variable (time of measurement) and two between-subject variables (conditions and sex) was conducted separately for PA and NA. Regarding NA, the results revealed no significant main or interactive effects. There was, however, a main effect of time, significant in the case of PA, *F*(1, 94) = 13.35, *p* < .001, $\eta_{\text{p}}^{2}$ = .12, in the rejection, but not the acceptance, condition. Although the interaction of time and manipulation was not significant (*p* > .12), PA decreased from *M* = 4.15, *SD* = 0.94, 95% CI [3.83, 4.49] to *M* = 3.61, *SD* = 1.51, 95% CI [3.17, 4.08] after same-sex rejection feedback, *p* = .002, and from *M* = 3.97, *SD* = 0.82, 95% CI [3.65, 4.32], to *M* = 3.45, *SD* = 1.10, 95% CI [2.98, 3.91] after opposite-sex rejection feedback, *p* = .003, but did not significantly change due to the acceptance manipulation (*p* > .75).

**Figure 1.**
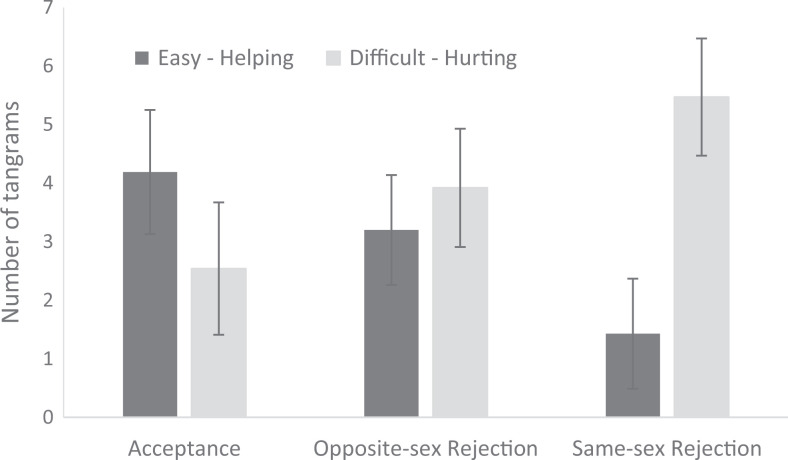
Means and 95% confidence intervals for helping (number of easy tangrams greater than 1) and hurting (number of difficult tangrams greater than 1) in experimental conditions.

The second hypothesis regarded differences in behavior due to the rejection manipulation and participants’ sex. The prediction was tested using a MANOVA with a 3 (same-sex rejection, opposite-sex rejection and acceptance) × 2 (man and woman) design because helping and aggression were highly correlated. Results show that the main effect of manipulation, Wilks’ λ = 0.83, *F*(4, 186) = 4.34, *p* = .002, $\eta_{\text{p}}^{2}$ = .09, and the main effect of participants’ sex, Wilks’ λ = 0.80, *F*(2, 93) = 4.34, *p* < .001, $\eta_{\text{p}}^{2}$ = .19, were significant. The interaction of conditions with participants’ sex was not significant (*F* < 0.43, *p* > .78, $\eta_{\text{p}}^{2}$ = .009). Next, we analyzed the significant multivariate effects separately for the helping and aggression scores. The main effect of manipulation was significant for aggression, *F*(2, 94) = 7.60, *p* = .001, $\eta_{\text{p}}^{2}$ = .14, as well as for helping, *F*(2, 94) = 8.05, *p* = .001, $\eta_{\text{p}}^{2}$ = .15. Helping was the lowest in the same-sex rejection, medium in the opposite-sex rejection, and the highest in the acceptance conditions. The reverse pattern of results was obtained for aggression. Regarding helping, post hoc Bonferroni tests showed that participants in same-sex rejection conditions differed from those in the acceptance condition, *p* = .001. Also, the same-sex rejection condition differed from opposite-sex rejection, *p* = .028, while the opposite-sex rejection and acceptance conditions were not different in this respect (*p* > .51). Post hoc tests analyzing the simple effects for aggression showed that it was higher in the same-sex rejection than in the acceptance condition (*p* = .001), but there was no significant difference between the same- and opposite-sex rejection conditions (*p* > .09; the difference was significant without Bonferroni correction, *p* = .03), and the opposite-sex rejection condition did not differ from the acceptance condition (*p* > .21). The main effect of participants’ sex was significant for aggression, *F*(1, 94) = 18.44, *p* = .001, $\eta_{\text{p}}^{2}$ = .16, but fell short of significance in the case of helping, *F*(1, 94) = 3.88, *p* = .052, $\eta_{\text{p}}^{2}$ = .04. Aggression was higher in men, *M* = 5.28, *SD* = 3.45, 95% CI [4.39, 6.17], than in women, *M* = 2.67, *SD* = 2.90, 95% CI [1.86, 3.46], while helping was higher in women, *M* = 3.50, *SD* =2.88, 95% CI [2.74, 4.26], than in men, *M* = 2.38, *SD* = 3.10, 95% CI [1.54, 3.21]. Means of belonging, helping, and aggression in experimental conditions are presented in Table 1 and in Figure 1.

**Table 1.** Means, Standard Deviations (*M*/*SD*) and 95% Confidence Intervals, of Belonging and Behavior.

Variables	Acceptance	Same-Sex Rejection	Opposite-Sex Rejection
	*M*/*SD*; [95% CI]	*M*/*SD*; [95% CI]	*M*/*SD*; [95% CI]
Belonging	6.00/1.01; [5.58, 6.52]	4.31/1.27; [3.89, 4.72]	3.62/1.38; [3.23, 4.07]
Helping	4.19/3.15; [3.13, 5.24]	1.43/2.00; [0.49, 2.36]	3.20/3.29; [2.26, 4.15]
Aggression	2.54/2.73; [1.41, 3.66]	5.47/3.43; [4.47, 6.46]	3.92/3.42; [2.91, 4.93]

## Discussion

Previous research has mainly studied rejection in a same-sex relationship and has not considered the sex composition of the rejectee–rejecter pair (Ayduk et al., 2008; Buckley et al., 2004; Warburton et al., 2006). This study tested the differences in affect, needs, and social behavior after rejection experienced in the context of same-sex and opposite-sex interactions. It was predicted that distress would be higher in rejection than in inclusion conditions, both among men and women, but that same-sex rejection would result in more distress than opposite-sex rejection. The results only partially confirmed the first hypothesis. Consistent with the findings of previous research (Blackhart et al., 2009), the study showed that the affect after rejection was less positive (but not more negative) than before rejection, while no such change was observed due to acceptance. Also, belonging need satisfaction was lower in both rejection conditions than in the acceptance condition. Those results confirm the first part of the hypothesis; however, no differences in affect or needs satisfaction due to the conditions emerged. Thus, it cannot be concluded that opposite-sex rejection resulted in less distress than same-sex rejection, as at least one other study found (Bolling et al., 2012). The findings of the present study suggest that rejection affects mood and needs equally among men and women in the same-sex and opposite-sex contexts, which is in accordance with the results of the meta-analysis by Hartgerink, Van Beest, Wicherts, and Williams (2015) of sex differences in the effects of ostracism on affect and needs but also Williams’s (2009) temporal need threat model of exclusion. The latter stresses the importance of noticing all, even slight rejection cues for survival. Thus, lowered positive affect and belonging need satisfaction may alarm the individual to cope immediately with a rejection threat.

Basing on the sexual exchange theory (Baumeister & Vohs, 2004) and the evolutionary conceptualization of aggression (Ferguson & Beaver, 2009; Gottschalk & Ellis, 2009) and rejection (Kurzban & Leary, 2001), we considered aggression, but also helping, as constituting strategies that allow for coping with rejection. We hypothesized that aggression would be higher and helping would be lower in the same-sex rejection than in the opposite-sex rejection and inclusion conditions, but the effect for aggression would be stronger in men and the effect for helping would be stronger in women. The results were in line with the hypotheses but only regarding the main effects of conditions. Same-sex rejection resulted in more aggression and less helping than opposite-sex rejection, but sex did not moderate that effect. The result corresponds to the sexual exchange theory assumption that sex is a resource with a current market price that can be exchanged for other resources. A same-sex person is perceived as a rival for resources (women’s sex in the case of men and men’s care, commitment, and assistance in case of women), while the opposite-sex other could be a potential customer or seller. Thus, aggression toward and less helping given to the same-sex rejecter could be good strategies of dealing with the consequences of rejection (lowered social status) but not so with opposite-sex rejection. Our study showed that people chose the most adaptive, or profitable from the economic point of view, strategy in each situation—more aggression and less helping behavior toward a same-sex rejecter and more neutral behavior toward an opposite-sex rejecter. In the case of both types of behavior, no differences were observed between acceptance and opposite-sex rejection, which clearly indicates that the most typical aggressive reaction to rejection (e.g., Leary et al., 2006) was in this case inhibited, as it could be too costly. It should also be noted that, although people combined both strategies for regaining their social status after the same-sex rejection, they relied more on limiting helping than on increasing aggression; this again suggests that people calculated the costs of each strategy in the particular situation, and it supports Hawley’s (1999) assumptions regarding differential preferences for dominance strategies.

The main effect of conditions—being less helpful and more aggressive toward the same-sex rejecter comparing to opposite-sex rejecter—could also be explained in light of the recalibration theory of anger (Sell, 2011). This model employs the concept of the welfare trade-off ratio (WTR; Tooby, Cosmides, Sell, Liberman, & Sznycer, 2008), which describes how much weight an individual should put on the welfare of another compared to themselves when making decisions that affect them both. The recalibration model posits that an individual will raise their WTR toward another; when doing so would be less costly than maintaining the lower WTR and bearing the costs of conflict and losing of cooperation possibilities. The theory also indicates that people negotiate their WTRs by increasing their ability to impose costs and confer benefits. Thus, people assess traits relevant to the ability to impose costs (e.g., aggression) and bestow benefits (e.g., mating value). Rejection usually increases anger (Chow, Tiedens, & Govan, 2008), and anger in recalibration theory is the result of an adaptation designed by natural selection that functions to recalibrate responses to another individual who has demonstrated lower than acceptable WTR toward the angry person. Imposing costs on the target of anger might make the target raise their WTR. However, although this may be a good strategy in the case of same-sex targets, it would be less beneficial in the case of opposite-sex targets since more weight is placed on the welfare of individuals who can efficiently bestow benefits. Opposite-sex others could be potential mates, thus, in case of opposite-sex rejecters, less aggressive WTRs negotiating tactics should be employed. Our study conformed with Sell’s model, as more aggression and less helping behavior was a response to rejection—a sign of low WTR toward the rejected person—but this cost infliction referred only to same-sex rejecters but not opposite-sex rejecters.

This study did not confirm predictions regarding sex differences in same- and opposite-sex rejection effects for behavior. We stated this hypothesis mainly on the basis of Gottschalk and Ellis’s (2009) review of evolutionary theories of aggression. They suggest that men compete more with other men and tend to use more risky strategies of gaining domination while doing so, such as aggression, whereas women compete less because they are more interested in finding high-quality mates who can provide resources. However, we also acknowledged, based on the sexual exchange theory, that although not as competitive as men, women still might compete with same-sex others but using less risky strategies of behavior, such as choosing not to help. Although previous studies on rejection effects did not show sex differences in aggression (e.g., Ayduk et al., 2008; Chow et al., 2008) or helping (e.g., Twenge et al., 2007), we assumed that measuring both hurtful and helpful behavior could allow for strategy choosing and that sex differences could become visible. The results contradicted our prediction and confirmed that responses to rejection are not different among men and women. However, the present study also showed that, irrespective of the experimental condition, men in general were more aggressive and less helpful than women. Such a result is also in agreement with Ferguson and Beaver’s (2009) conceptualization of aggression as a more adaptive strategy of achieving dominance for men. Similar general tendencies were also indicated in other studies with respect to aggression (Archer, 2004) and helping (Bierhoff, 2002).

### Other Possible Interpretations of the Results

Although our predictions were based on an evolutionary perspective, it must be acknowledged that processes related to group identity could also play an important role in reactions to rejection in same-sex and opposite-sex relationships. In this case, same-sex ostracism could be conceptualized as an intragroup behavior and opposite-sex ostracism as an out-group behavior (Bolling et al., 2012). In another study based on the same assumptions, but testing for same/different-race rejection, Burnstein, Sacco, Young, Hugenberg, and Cook (2010) argue that, because of in-group favoritism, rejection by a member of a group that is essential for social identity formation and maintenance (e.g., same-race or same-sex) should be more painful. Also, rejection by an in-group member is more powerful because it does not allow attribution of the causes of rejection to the rejecter’s out-group status. For example, a man rejected by a woman could speculate that he was rejected because he was not a woman. Indeed, Bolling et al. (2012) found more pronounced distress and a more negative emotional response in exclusion by same-sex others than in exclusion by opposite-sex peers. This line of argument has not found confirmation in our study, as the belonging threat or negative affect was not higher under the same-sex than under the opposite-sex condition. Taking into account the inconclusive results of studies testing the effect of same- and other-group identities, such as race or political preferences, on distress after exclusion (Bernstein, Sacco, Young, Hugenberg, & Cook, 2010; Gonsalkorale & Williams, 2007; Krill & Platek, 2009; Paolini, Alparone, Cardone, vanBeest, & Merla, 2016; Smith & Williams, 2004), the group identification issue should be considered in future research.

## Limitations and Future Research

We are also aware of the study’s limitations. As the main hypothesis predicted an interaction between the sex of the rejectee and being rejected by the same and opposite-sex other, it is important to obtain adequate statistical power for testing such an interaction; one could speculate that the lack of interactive effect of participants’ sex and manipulation resulted from insufficient power in our study. Indeed, post hoc power analysis given α = .05, sample size and effect size ( $\eta_{\text{p}}^{2}$ = .002 for aggression and $\eta_{\text{p}}^{2}$ = .012 for helping), indicated very low power of this study to detect a significant interaction effect, 14% in case of helping and only 6% in case of aggression. Given the very low effect sizes, at least 1,275 participants would be needed to detect an interaction effect for helping and many more for aggression. Although it would be difficult to obtain such large sample sizes, replication of the study with more participants should be conducted to confirm that men and women do not differ significantly in their behavioral reaction to same-sex and opposite-sex rejection.

Future research should also include information on the partner’s sex in acceptance conditions, allowing for comparison of same-sex and opposite-sex acceptance but should also add a fully neutral condition to separate the effects of exclusion and same/opposite-sex conditions for behavior. Also, our protocol did not make the mating potential in the opposite-sex condition salient, so other studies might include information that the opposite-sex rejecter could be a possible mating partner (e.g., for a date). Nevertheless, the study should also be replicated with a different approach to rejection manipulation and aggression/helping measurement.

Another interesting question refers to individual differences which could shape reactions to same- and opposite-sex rejection. As Romero-Canyas et al. (2010) posit, personality characteristics and other contextual factors could also influence the behavior of men and women after rejection in same-sex and opposite-sex conditions. Thus, including, for example, rejection sensitivity (Downey & Feldman, 1996) or sex group identification measures could bring new insights into the results obtained in this study. We also suggest focusing on mechanisms behind the same/opposite-sex effect. At present, it has not been determined what stands behind more negative behavioral reactions in the same-sex rejection condition. It is still to be resolved whether it is the in-group/out-group effect, or the same-sex competition/opposite-sex resource exchange effect, or both.

## Conclusions

The findings of this study shed some light on the role that the sex of the rejecter plays in the emotional and behavioral responses of men and women. The original value of the study was that it allowed for comparison of men’s and women’s positive and negative behavior toward same-sex and opposite-sex rejecters. The study confirmed the prediction that, after rejection, people, irrespective of their sex, behave less aggressively and are more helping toward the opposite-sex other than toward the same-sex other; we explain our results in line with the evolutionary approach, acknowledging motivation to compete with same-sex individuals and the motivation to exchange resources in opposite-sex situations even under rejection conditions. The obtained effect could also be understood as an effect of attribution of causes for rejection to other-group identity, making the rejection less powerful in the opposite-sex situation. Our study’s secondary contribution is to provide another look at the general negative emotional response to social rejection in the context of sex differences. Rejection caused a drop in positive affect and belonging, but emotions and needs were not influenced by the same/opposite-sex condition or by the sex of the rejectee, confirming the assumption of Williams’s (2009) need threat model, that the immediate reaction to rejection is social pain, which is not shaped by sex or other situational conditions.
